# Preparation of a Dual-Functional Active Film Based on Bilayer Hydrogel and Red Cabbage Anthocyanin for Maintaining and Monitoring Pork Freshness

**DOI:** 10.3390/foods12244520

**Published:** 2023-12-18

**Authors:** Xiaowei Huang, Wanying Zhao, Zhihua Li, Ning Zhang, Sheng Wang, Jiyong Shi, Xiaodong Zhai, Junjun Zhang, Tingting Shen

**Affiliations:** 1 School of Food and Biological Engineering, School of Agricultural Equipment Engineering, Jiangsu University, 301 Xuefu Rd., Zhenjiang 212013, China; huangxiaowei@ujs.edu.cn (X.H.); 15506509723@163.com (W.Z.); zhangning980409@163.com (N.Z.); 19825822627@163.com (S.W.); shi-jiyong@ujs.edu.cn (J.S.); zhai_xiaodong@ujs.edu.cn (X.Z.); jjzhang95@126.com (J.Z.); shentingtingstt@ujs.edu.cn (T.S.); 2 College of Food Science and Engineering, Nanjing University of Finance and Economics/Collaborative Innovation Center for Modern Grain Circulation and Safety, 128 North Railway Street, Gulou District, Nanjing 210023, China; 3 Focusight (Jiangsu) Technology Co., Ltd., No. 258-6 Jinhua Road, Wujin Economic Development Zone, Changzhou 213146, China

**Keywords:** active and intelligent packaging, bilayer hydrogel, red cabbage anthocyanin, antibacterial activity, freshness monitoring, pork

## Abstract

In this study, a composite film was created with the dual goal of prolonging pork shelf life and showing freshness. Hydrogel materials as solid base films were selected from gelatin (G), sodium alginate (SA) and carboxymethyl cellulose (CMC) based on their antioxidant activity, water vapor permeability, mechanical properties, as well as their stability, antimicrobial activity, and freshness, which indicates effectiveness when combined with anthocyanins. Furthermore, the effects of several concentrations of red cabbage anthocyanin (R) (3%, 6%, 12%, and 24%) on freshness indicators and bacteriostasis were investigated. The antimicrobial activity of the composite films was evaluated against *Escherichia coli*, *Bacillus subtilis*, and *Staphylococcus aureus*. Likewise, the freshness indicates effectiveness was evaluated for NH_3_. Considering the mechanical properties, antibacterial ability, freshness indicator effect, and stability of the composite film, CS film combined with 12% R was selected to prepare a dual-functional intelligent film for pork freshness indicator and preservation. By thoroughly investigating the effect of composite film on pork conservation and combining with it KNN, the discriminative model of pork freshness grade was established and the recognition rate of the prediction set was up to 93.3%. These results indicated that CSR film can be used for the creation of active food packaging materials.

## 1. Introduction

Meat is rich in nutrients and prone to microbial growth, so monitoring and maintaining the freshness of meat has always been a concern for consumers and businesses [1]. Experience-based predetermined expiration dates cannot adapt to the increasingly complex meat logistics model, which might result in the misuse of perishable food or food wastage [2]. According to the UN Food and Agriculture Organization, 1.3 billion tons of food worth more than USD 1 trillion end up lost or wasted every year, accounting for one-third of global food production. More than half of food loss and waste is caused by spoilage. At the same time, 870 million people still struggle to meet their daily needs, and one in three children suffers from malnutrition [3]. Therefore, it is equally important to develop safe meat antibacterial agents and online monitoring technologies for meat freshness. Intelligent food packaging is one of the new packaging methods that have emerged in recent years. It can inform consumers of the food’s freshness by monitoring some characteristics or environmental parameters of the food inside the packaging, and it can also extend the shelf life of food by utilizing the active substances attached to the packaging [4]. Meat spoilage is mainly caused by microbial reproduction. During this process, microorganisms metabolize proteins to produce nitrogen-containing volatile substances, collectively called total volatile basic nitrogen (TVBN). It is an important indicator for evaluating the freshness of meat. As the degree of meat spoilage increases, the increase in TVBN content leads to an alkaline environment in the packaging [5]. Therefore, freshness detection can be achieved through colorimetric sensing films that rely on changes in pH, as well as by inhibiting microbial reproduction to maintain meat freshness. Many plant extracts have been shown to have antibacterial and antioxidant properties owing to their high levels of phenolic components, such as flavonoids, phenolic acids, and tannins [6,7,8,9]. These plant extracts have the benefits of being derived from many sources, safety, and non-toxicity, and having a cheap cost of extraction. Among them, anthocyanins have been found to assess the freshness of meat products by detecting changes in color caused by variations in the pH level of the gas within the packaging. This allows customers to get real-time information regarding the freshness of the meat products [10]. Hence, anthocyanins serve as an optimal antibacterial agent and indication of freshness for meat products. However, its chemical property is inherently unstable and prone to easy decomposition [11]. Studying suitable substrate materials is essential to guarantee the stability of anthocyanins as indications of antibacterial properties and freshness in meat products. Gelatin, sodium alginate, and carboxymethyl cellulose are biodegradable materials that are well-suited for food packaging due to their excellent film-forming capabilities, high plasticity, and robust biocompatibility. When used as substrates for film formation, they have the potential to enhance the mechanical characteristics, physicochemical properties, and barrier properties. Additionally, they may protect the stability and active functions of active components in intelligent packaging.

In order to obtain a dual-function method of pork freshness preservation and indication, a simple and low-cost colorimetric fiber film was created by embedding beads on a hydrogel substrate and red cabbage anthocyanin. Hydrogel materials as solid base films are selected from gelatin, sodium alginate, and carboxymethyl cellulose based on their antioxidant activity, water vapor permeability, mechanical properties, stability, antimicrobial activity, and effectiveness in indicating freshness when combined with anthocyanins. The concentrations of red cabbage anthocyanin were investigated for their antibacterial and freshness indicating properties. Ultimately, the resulting composite film was employed as an intelligent packaging material for coating pork to verify its freshness preservation and indication function.

## 2. Materials and Methods

### 2.1. Materials

The pork loin was provided by a slaughter plant (Fengyuan Co., Zhenjiang, China) within one hour after slaughter in insulated polystyrene boxes on ice. Red cabbages were provided by Chunlei Huacao Tea (Haozhou, China) Co., Ltd. Hydrophobic microporous filter film, qualitative filter paper, and a spotting capillary tube (100 mm × 0.5 mm) were provided by Huadong Instrumented Glass (Zhenjiang, China) Co., Ltd. *Escherichia coli* (CICC 20658), *Bacillus subtilis* (CICC 10732), and *Staphylococcus aureus* (CICC 10201) were purchased from the China Center for the Preservation and Management of Industrial Microbial Cultures. Ethanol, citric acid monohydrate, potassium chloride, sodium acetate, concentrated hydrochloric acid, and LB medium were purchased from Sinopharm (Shanghai, China) Co., Ltd. 1,1-Diphenyl-2-picrylhydrazyl (DPPH) was purchased from Sigma-Aldrich Co., Inc (St. Louis, MO, USA).

### 2.2. Preparation of Composite Films

**Figure 1:**
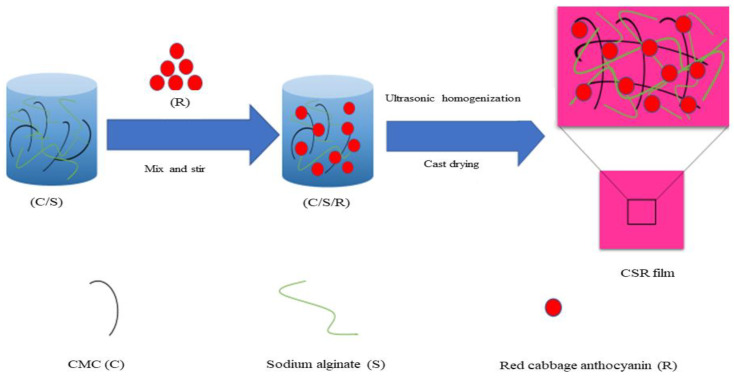
Scheme of preparation for composite films.

The red cabbages were washed and dehydrated at ambient temperature. Subsequently, the 20-g powder was introduced into 100 mL of ethanol 80% (*v*/*v*). The mixture was stirred in a water bath for 30 min at 50 °C. Gelatin powder was dissolved in distilled water and heated to 60 °C then constantly stirred to create a gelatin solution (4%, *w*/*v*). Then, sodium alginate powder (3% *w*/*w*) was slowly added to the gelatin solution in a water bath at 45 °C for 20 min. The carboxymethyl cellulose solution (2% *w*/*v*) was prepared by dissolving carboxymethyl cellulose to distilled water heated at 45 °C with continuous stirring until fully dissolved. Afterward, six different bioactive mixed solution samples were prepared by adding red cabbage anthocyanin (R) to three different solutions, which were mixed at 1:1 volume with glycerol (1% *v*/*v*). All solutions were mixed by using the Ultrasonic Cleaner at 50 w and bubbles were removed by degassing. Using Petri dishes with a 90-mm diameter, the composite films were created using the solvent-casting method during a period of 36 h at 35 °C. The scheme of preparation for composite films is shown in Figure 1.

### 2.3. Characterization of Composite Films

#### 2.3.1. The Color Change of Films

The color variation of films (pH 2–12) was observed and photographed.

#### 2.3.2. The Color of Composite Films

The composite films were evaluated using a colorimeter and the CIEAB color scale. The parameters measured were *L* (brightness), *b* (-blue to +yellow), *a* (-green to + red), and the color difference (Δ*E*) [12]. The following equation was used to determine this parameter:

(1) $$△E=\sqrt{\left[(L-L_{0}{)}^{2}+(a-a_{0}{)}^{2}+(b-b_{0}{)}^{2}\right]}$$

The measured values of the samples to be tested are represented by *L*, *a*, and *b*, while *L*0 (91.13), *a*0 (−0.76), and *b*0 (3.58) correspond to the color parameters of a white plate used for calibration.

#### 2.3.3. Light Transmission and Opacity

Using a UV/VIS spectrophotometer (Beijing Ruili Analytical Instruments Co., Ltd., Beijing, China), the absorbance at the wavelength of 600 nm was measured to determine the opacity [13]. It was calculated using Equation (2) as follows:

*Opacity* = *Abs*_600_/*Th* (2)

where *Th* is the thickness of each film (mm) and *Abs* 600 is the absorbance at 600 nm.

#### 2.3.4. Thickness and Mechanical Properties

A digital micrometer was used to measure the thickness of composite films. Thickness data were measured at six locations on the films and represented as an average (μm). The mechanical properties of the samples were determined by the specifications stated in GB 13022-1991 [14]. Samples measuring 6 mm × 2 mm were fabricated using the texture analyzer TA-96. Following that, at a temperature of 25 °C and 45% relative humidity (RH), the tensile strength (*TS*) and elongation at break (*EB*%) were determined using an elongation speed of 0.6 mm/s and an initial film length of 40 mm [2]. Before conducting the tests, the samples were cut into 2 mm × 2 mm squares. The traction speed was consistently maintained at 1 mm/s while starting with an initial length of 30 mm, and the maximum force (*F_max_*) was recorded [14]. The subsequent equation was employed to derive the results:

(3) TS=F/w×Th

(4) $$EB=L_{1}/L_{0}\times 100$$

(5) $$PS=F_{max}/Th$$

where *F* (N) is the tensile strength at fracture; the width of the film (mm) is denoted by *w*, while the thickness of the film (mm) is denoted by *Th*; *L*_1_ (mm) and *L*_0_ stand for the original film lengths and the lengths at which the stretched fibers snapped, respectively; *PS* is the puncture strength, and *F_max_* is the maximum puncture force.

#### 2.3.5. Moisture Content (MC) and Water Solubility (WS)

The MC values of the composite films, assessed in three identical samples, were obtained to establish the starting weight (*m_i_*). Subsequently, the film samples were dried at 105 °C in an oven for 24 h to obtain (*m_f_*). MC was determined using Equation (6) as follows:

(6) $$MC(\%)=\frac{m_{i}-m_{f}}{m_{i}}\times 100\%$$

where *m_i_* is the starting sample weight (g) and *m_f_* reflects the weight following a 24-h oven period (g).

The *WS* was approximated using a technique that was previously described [15]. After drying, every dried sample was submerged in water at 25 °C for 24 h. The water solubility of composite films was determined by taking three parallel samples. It was determined using Equation (7), which is listed below:

(7) $$WS(\%)=\frac{W_{i}-W_{f}}{W_{i}}\times 100$$

where *W_i_* represents the weight of the film after submersion and *W_f_* represents the weight after drying.

#### 2.3.6. Water Vapor Permeability (WVP)

The *WVP* was assessed using a previously described approach [16]. Three random samples were selected from each composite film. The samples were placed in a desiccator set at 25 °C and 0% RH. Each sample contained 20 mL of distilled water and was sealed in centrifuge tubes. Values were recorded six times daily, up to a maximum duration of 72 h, as documented in reference [17]. The *WVP* value was derived using the equation:

(8) $$WVP=\frac{\Delta m\cdot Th}{S\times \Delta P\times t}$$

where Δ*m* is the changes in the test vial weight (g); *Th* represents the thickness of each film (mm); *S* is the exposed area of each film (m^2^); *t* represents the duration (h); and Δ*P* is the 3167 Pa at 25 °C.

### 2.4. Antioxidant Capacity of the Film

Film samples (20 mm × 20 mm) were added to 4 mL of methanol at 25 °C in the dark for 2 h. The 3 mL sample was extracted and combined with 1 mL of 150 mol/L *DPPH* in methanol. For 30 min, the mixture was left at 25 °C in the dark. A spectrophotometer was used to measure the mixture’s absorbance at 517 nm [18]. The DPPH radical scavenging activity was calculated by the equation:

(9) $$\mathrm{DPPH}\;\mathrm{scavenging}\;\mathrm{activity}\;(\%)=\frac{A_{0}-A_{1}}{A_{0}}\times 100$$

where *A*_0_ represents the absorbance of DPPH in the control and *A*_1_ corresponds to the absorbance of the film.

### 2.5. Antimicrobial Activity of Composite Films

To assess the antibacterial characteristics of composite films, *Escherichia coli* (CICC 20658), *Bacillus subtilis* (CICC 10732), and *Staphylococcus aureus* (CICC 10201) of various strains were chosen as typical representatives of spoilage organisms in meat products. Moreover, Staphylococcus aureus and Bacillus subtilis are typical representative of Gram-positive (G^+^) bacteria while Escherichia coli is a typical representative of Gram-negative (G^−^) bacteria. The 100 μL, 105 CFU/mL bacterial suspension was completely immersed in a 6 mm-diameter film sample for 12 h. The rest of the bacteria were then grown for 12 h at 37 °C in 900 μL of LB medium. Ultimately, bacterial suspension (500 μL) was measured for optical density (OD) using a 600 nm-based UV-visible spectrophotometer. The bactericidal ratios of the films were determined using the formula below:

(10) $$Antibacterial\;ratio=\frac{OD\;of\;control-OD\;of\;test\;film}{OD\;of\;control}\times 100\%$$

In order to observe the antibacterial effect more intuitively, the film samples were attached to the culture medium and placed in a 37 °C incubator for 24 h [9]. The vernier scale was used to measure and calculate the diameter of the bacteriostatic ring, which was measured by the cross-measurement method.

### 2.6. Colorimetric Response and Color Stability

We used the technique described by J. Wang et al. [16] with some minor changes. First, the film (20 × 20 mm) was positioned 1 cm above the solution container for 20 min with 80 mL in a 500 mL box of an 8 mmol/L ammonium solution [19], then the images of the films were captured by a Canon 70D camera and analyzed by a self-compiled program in MATLAB 2019b. The color change was calculated from the Euclidian distance among the *RGB* intensities of the sample and reference images using the following equation:

(11) $$S_{RGB}=\frac{\left|R_{a}-R_{b}\right|+\left|G_{a}-G_{b}\right|+\left.{\left|B_{a}-B\right.}_{b}\right|}{R_{b}+G_{b}+B_{b}}\times 100\%$$

where *Rx*, *Gx*, and *Bx* show the standard deviation values of the red, green, and blue, respectively, and the subscripts *b* and *a* indicate the before and after color change, respectively. Additionally, the sum of the squares of the *RGB* deviation was used to calculate the square root of the real color uniformity of the samples [20].

The film (20 × 20 mm) was placed in a PE bag and stored at 4 °C and 25 °C for a period of 16 days. The stability of the films was determined by calculating the overall rate of change of RGB values extracted from the pictures [15].

### 2.7. Infrared Spectroscopy and Characterization of Microstructure

The attenuated total reflectance FT-IR spectrum of the film sample was analyzed using a Varian 670 spectrometer (Varian Inc., Palo Alto, CA, USA). The scanning frequency was set at 32 scans, with a resolution of 4 cm^−1^, and the measurement range was 600–4000 cm^−1^.

The surface morphology of films was analyzed using a scanning electron microscope. Prior to analysis, samples were coated with gold in a sputtering device and looked at a 15 kV accelerating voltage.

### 2.8. Application for the Pork Freshness

#### 2.8.1. Determination of pH

Samples were prepared by cutting up 10 g of pork, adding 100 mL of distilled water to a beaker, mixing the samples using a disperser, then letting it remain for 30 min. Finally, the ultimate pH of the leachate was measured.

#### 2.8.2. Determination of Lipid Oxidation

Thiobarbituric acid (TBARS) assay was used to assess the degree of lipid oxidation found in samples of pork. The 5 g of pork samples were homogenized after being dispersed in 7.5% trichloroacetic acid. The homogenate was filtered using filter paper, and then the filtrate was heated to boiling (100 °C) for 40 min before being reacted with 0.02 M TBARS. The 532 nm absorbance measurement was made from the pink supernatant.

#### 2.8.3. Determination of Total Viable Count (TVC)

Pork samples weighing 10 g were sliced, combined with 10 mL of sterile saline, and then subjected to three gradients of dilution at a ratio of 1:10. In a sterile environment, 0.1 mL of diluent solution was coated with the petri dish, which was then kept at 37 °C for 48 h in the incubator.

#### 2.8.4. Determination of TVB-N

The pork meat (10 g) was minced and homogenized in 50 mL of distilled water. Then, after pouring the liquid into a distillation tube, 5 mL of MgO (10 g/L) were added [21]. Next, 10 mL of 2% boric acid was added as the recipient solution. The solution was treated with HCl (0.01 mol/L) until it reached a blue-purple color after the reaction was finished. The following is the TVB-N content calculation formula:

(12) $$X=\frac{(V_{1}-V_{2})\times c\times 14}{m\times 5/100}\times 100$$

where *V*_1_ (mL) is the amount of HCl absorbed by the sample, *V*_2_ (mL) is the amount swallowed by the control group, *C* (M) is 0.01 mol/L, and *m* (g) is the weight of the pork samples. *X* represented the pork TVB-N content in (mg/100 g).

### 2.9. Statistical Analysis

The film data were analyzed using the Statistical Package for the Social Sciences (SPSS) Statistics 27 software. The results were reported as means ± standard error. The significance level was set as a *p*-value less than 0.05.

## 3. Results and Discussion

### 3.1. The Color of Composite Films

#### 3.1.1. The Color of Untreated Composite Films

Packaging color qualities play an important role in terms of overall appearance and consumer acceptance [22]. Table 1 displays alterations regarding the rectangular coordinates (*L*, *a*, and *b*), indicating that these color attributes exhibit a consistent trend. Furthermore, an increase in red cabbage anthocyanin content leads to a significant modification in the L, a, and b values of the films (*p* ≤ 0.05). Upon the addition of red cabbage anthocyanin, a decrease in a value was seen, resulting in the green appearance of the GSR and GCR films. Conversely, the CSR film exhibited a substantial rise in value, leading to a red coloration of the film. These findings indicate that gelatin is an alkaline polysaccharide, and that the pH level affects the color of red cabbage anthocyanin [23,24]. Nevertheless, the b parameter exhibits a significant disparity. Unlike the CS film, the GS and GC films exhibited elevated levels of b and opacity, which may be attributed to the gelatin film’s mild yellow hue and high opacity. Subsequently, the CS and GC films exhibited the most elevated and lowest measurements of the opacity parameter (2.20 and 0.51, respectively), as a result of the impact of the films’ internal structure [25].

#### 3.1.2. The Color Change of Composite Film

The color of the composite film in buffer solution (pH 2.0–12.0) is shown in Figure S1. The color of the film was pink within pH 2.0–7.0, green at pH 8.0, purple at pH 9.0, and yellow between pH 10.0–12.0. The result indicates that the change in color may be attributed to the diverse molecular composition of anthocyanin.

### 3.2. Properties of Composite Indicator Film

#### 3.2.1. Infrared Spectrum Analysis of Composite Film

**Figure 2:**
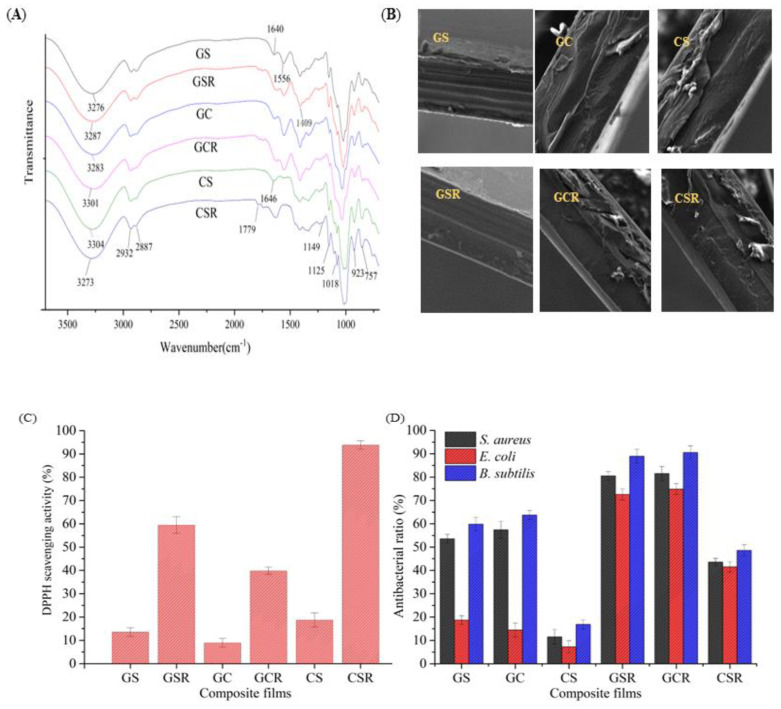

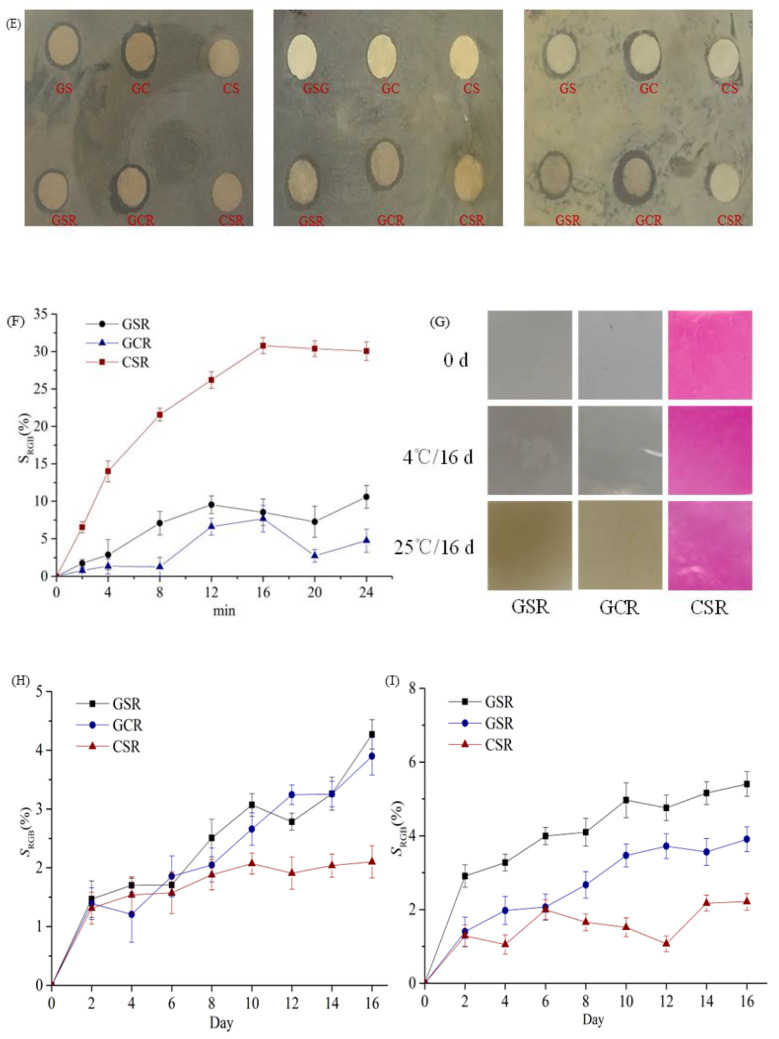
Properties of composite indicator film: (**A**) FTIR spectra of composite films; (**B**) Scanning electron microscope (SEM) pictures of composite films (Magnifications 1000×); (**C**) Antioxidant ability of composite films; (**D**) Antibacterial ratio of the intelligent active films to *S. aureus*, *E. coli* and *B. subtilis*; (**E**) Photos of composite film forming solution antimicrobial test against; (**F**) Composite films response toward ammonia vapor; (**G**) Photos of composite films at 4 °C and 25 °C after 16 d; (**H**) Stability of composite films at 4 °C; (**I**) Stability of composite films at 25 °C.

The FT-IR spectra of the composite films are shown in Figure 2A. In the spectrum of gelatin, there is a strong absorption band with a maximum at 3273 cm^−1^ assigned to the -NH stretching vibration [24,26]. The addition of anthocyanins resulted in noticeable shifts in the peaks of the composite film, suggesting that the observed phenomenon could be attributed to hydrogen bonding interactions. The characteristic peak of gelatin exhibits a typical C=O (amide I) stretching band at 1640 cm^−1^, besides the band at 1556 cm^−1^ associated with the bent vibration of the N-H (amide Ⅱ) [27]. The introduction of red cabbage anthocyanin resulted in the appearance of additional peaks in the GSR, GCR, and CSR films (at 1775 cm^−1^, 1778 cm^−1^, and 1779 cm^−1^, respectively); these are attributed to the stretching vibration of ketone C=O. With the range of 1500 to 1600 cm^−1^, which corresponds to the stretching vibration of C=C aromatic rings, significant fluctuations are seen, indicating the presence of aromatic compounds in the red cabbage anthocyanin. The peaks at 2934 cm^−1^ and 2887 cm^−1^ correspond to the stretching vibrations of -CH_2_ and -CH, respectively [28]. The spectral region between 3500 cm^−1^ and 2800 cm^−1^ where bonding energy is enhanced leads to changes in the mechanical properties of the film due to the presence of adjacent aromatic ring substitutions [9].

#### 3.2.2. The Morphology and Structure

The spatial arrangement of various components and the drying process have an impact on the microstructure of the films [29]. The GS and GC films exhibit a smooth and uniform surface, suggesting a strong compatibility between gelatin and CMC/SA [30]. Nevertheless, the CS film exhibited a heterogeneous division in the cross-section, revealing the organized arrangement of some segments of polymer chains [31]. The incorporation of anthocyanins into the GCR film results in a textured and network-like arrangement, suggesting that the cohesive structure of gelatin/SA is disturbed. However, the CSR film shows a smooth surface and a dense structure, indicating that the red cabbage anthocyanin can enhance CMC/SA cooperation.

#### 3.2.3. Physical and Mechanical Properties

The thicknesses and mechanical properties of composite films was shown in Table 2. The thickness of the GS film was the smallest at 83.60 ± 5.47 µm. The addition of red cabbage anthocyanin led to a substantial increase in the thickness of the films. The GCR film exhibited a maximum thickness of 104.20 µm, which may be attributed to the properties of the substrates used for film formation. The TS and EB values were significant indicators as they were related to the handling, transportation, and storage methods of food packaging [32].

Incorporating red cabbage anthocyanin significantly enhanced the tensile strength. The GCR film had the highest TS values. This phenomenon might be attributed to the formation of polymer inter-chain hydrogen bonds between gelatin and CMC, which hinder the mobility of molecular chains in CMC that can rotate freely. Conversely, the inclusion of red cabbage anthocyanin led to a significant reduction in EB. However, the CSR film has a very high EB value of 88.71, which may be attributed to the presence of red cabbage essence. The biocompatibility of CMC and SA has been improved, resulting in more homogeneous film components and a more compact framework.

Table 2 shows the MC, WS, and WVP of the composite films. The CSR film showed an MC value of 16.52 ± 1.26 ^b^. This value was much lower than the pure CS film obtained with the MC value of 32.69 ± 0.38 ^a^ due to the phenolic hydroxyl group of the red cabbage anthocyanin. This group has a greater affinity for forming hydrogen bonds with the hydroxyl group of the matrix, hence reducing the extent of cross-linking between the composite films and the water molecules [33,34].

The data revealed that the presence of red cabbage anthocyanin increased the WS of the samples due to the polyphenolic compounds in the red cabbage anthocyanin weakening the hydrogen bonding force present in the film and promoting the film’s swelling ability.

Food packaging applications typically call for films with low water vapor permeability because preventing the microbiological and chemical deterioration of food systems is the primary objective of packaging materials [9,35]. The effect of red cabbage anthocyanin incorporation on the water vapor permeability of films is shown in Table 2. The water barrier property of the composite films was evaluated using the WVP. In general, food packaging is better suited to lower WVP [32]. The GS film showed the lowest WVP with an amount of 3.08 ± 0.04 ^c^ g m^−1^s^−1^Pa^−1^. Meanwhile, the WVP of the CS film is 6.02 ± 0.60 ^bc^ g m^−1^s^−1^Pa^−1^. According to Tabari et al. [36], the CMC film, perhaps as a result of the larger anionic side groups of CMC increasing the free volume of the composite matrix, is more likely to absorb water. Since the red cabbage anthocyanin is hydrophilic, it demonstrates that the film would be permeable to gas; the CSR film has shown a higher WVP.

#### 3.2.4. DPPH Radical Scavenging Activity

The antioxidant activities of composite films are assessed by measuring the percentage of DPPH scavenging, as shown in Figure 2C. Overall, when the amount of red cabbage anthocyanin was increased, the antioxidant activity of composite films considerably increased [9]. The potent antioxidant action is likely attributed to the polyphenol anthocyanins, which have a high concentration of phenolic hydroxyl groups. Phenolic hydroxyl groups counteract free radicals by producing phenoxy groups [37]. The GSR, GCR, and CSR films displayed distinct antioxidant activity, with a statistically significant difference (*p* ≤ 0.05). Gelatin apparently caused the GSR and GCR films to be formed in an alkaline environment, reducing the overall phenolic content of the films and resulting in lower antioxidant activity [38]. The release of active compounds and other factors, such as the interaction of active components with polymers and the microstructure of the films, was the key determinant of the ability of composite films to combat free radicals [39].

#### 3.2.5. Antimicrobial Activity

From Figure 2D, it can be seen that GS and GC film solutions show significant antibacterial effects (*p* ≤ 0.05). The reason for the antibacterial effect is that the gelatin components in GS and GC films solutions contain positively charged amino groups, which can damage the negatively charged microbial cell film, causing leakage of intracellular cytoplasm within the film and leading to bacterial death. The most significant antibacterial action was demonstrated by GC films, with bactericidal rates against *S. aureus*, *E. coli*, and *B. subtilis* reaching 57.45%, 14.45%, and 63.76%, respectively. This may be due to the oxygen barrier properties of CMC in the GC film solution, while *S. aureus*, *E. coli*, and *B. subtilis* are aerobic microorganisms that can synergize with gelatin to inhibit microbial proliferation [16]. After adding red cabbage anthocyanin, the antibacterial ability of all composite films improved, and the inhibitory effect of the CSR film solution on *S. aureus*, *E. coli*, and *B. subtilis* significantly improved (*p* ≤ 0.05). This indicates that red cabbage anthocyanins possess antibacterial activity and exhibit a synergistic effect. The GCR film solution achieved the most substantial inhibitory effect on *S. aureus*, *E. coli*, and *B. subtilis*, with antibacterial rates of 81.54%, 74.85%, and 90.54%, respectively, followed by the GSR film solution. The antibacterial assays (Figure 2E and Table S1) demonstrated the same antibacterial effect. Therefore, red cabbage anthocyanin holds significant promise for use in pork preservation to inhibit microbial growth.

#### 3.2.6. Volatile Ammonia Response

As observed in Figure 2F, films’ response to volatile ammonia can be employed to mimic the production of vaporous nitrogen molecules during the rotting of pork. With increasing time, the color and S_RGB_ of GSR, GCR, and CSR films changed with increasing NH_3_ concentration. These findings showed that ammonia caused an alkaline environment around the films, leading to the formation of chalcone from anthocyanins, which resulted in a color change in the films [33]. In contrast, the CSR film exhibited the highest sensitivity to ammonia. The CSR film exhibited the highest sensitivity to NH3, followed by the GSR film, and the least sensitivity was observed in the GCR film. The pH changes in the composite film of anthocyanins were caused by the alkaline environment. Sensitivity variations between the composite films, on the other hand, could be explained by the pH of the substrate used for film formation [33]. It was widely assumed that an indicator film would have a quick response time. As a result, the CSR film, with the highest rate of color shift, would be beneficial for its application.

#### 3.2.7. Stability of the Films

The stability of the film was also evaluated. As illustrated in Figure 2H,I, over 16 days, the composite films’ (S_RGB_) rate of color change was measured at 4 °C and 25 °C. The *S*_RGB_ increased significantly with a rise in temperature. The *S*_RGB_ value did not exceed 10% in 16 days, suggesting that the composite films had high stability. The stability test results obtained from the composite films under storage conditions of 4 °C and 25 °C were consistent. The stability of the stable CSR film was the strongest, followed by the GCR film, and then the GSR film.

### 3.3. Application of Composite Films for Pork Freshness Maintaining and Monitoring

#### 3.3.1. Determination of Concentration in Red Cabbage Anthocyanin

**Figure 3:**
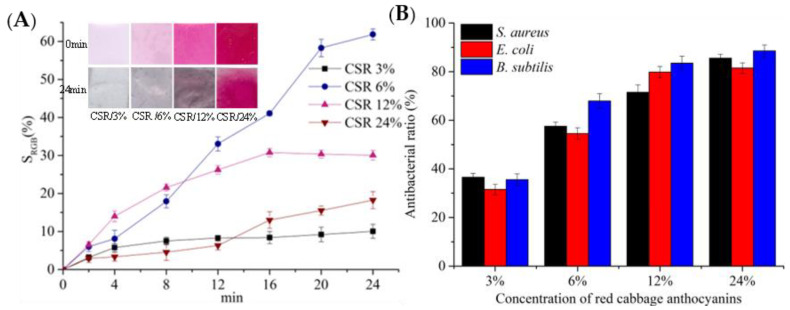
(**A**) Composite films with different concentration response toward ammonia vapor, (**B**) Antibacterial ratio of the intelligent active films with different concentration.

Based on the preceding research, it was found that the red cabbage anthocyanin significantly influences the microstructure of the composite films and is biocompatible with the carboxymethyl cellulose/sodium alginate matrix (CS). The composite film (CSR) made from red cabbage, carboxymethyl cellulose, and sodium alginate exhibits superior DPPH scavenging activity and a highly sensitive and stable NH_3_ response. Although the antibacterial activity of CSR is not satisfactory, its antibacterial activity can be enhanced by adjusting the red cabbage anthocyanin content. Therefore, CSR films were selected for future research. Various amounts of red cabbage anthocyanidin (3%, 6%, 12%, and 24% (*w/w*)) were incorporated into the CS film, yielding CSR films denoted as CSR/3%, CSR/6%, CSR/12%, and CSR/24%, respectively. Higher concentrations of red cabbage anthocyanidin result in a more pronounced bacteriostatic effect in the composite films (Figure 3B). In summary, a 3% concentration of red cabbage anthocyanidin led to the lowest ammonia response sensitivity and bacteriostatic effect in the composite films. At a 24% concentration of red cabbage anthocyanidin, the bacteriostatic effect was maximized, albeit with a minimal NH_3_ response. Subsequent experiments employed composite films with 6% and 12% red cabbage anthocyanidin concentrations, focusing on further exploration of the optimal anthocyanin concentration in composite films.

#### 3.3.2. Sample Preparation

**Figure 4:**
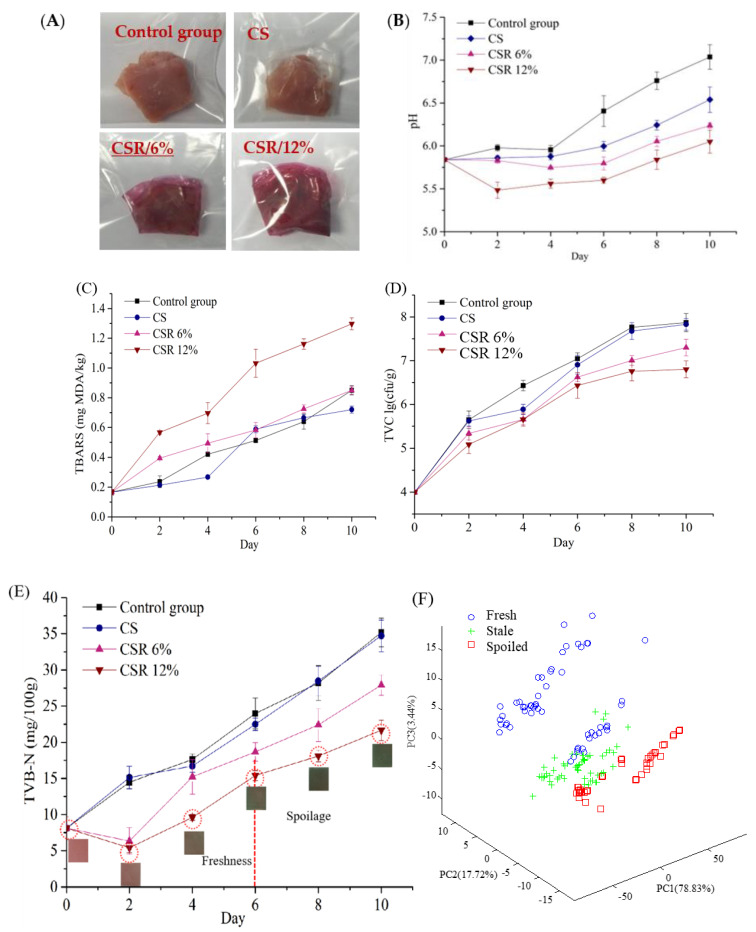

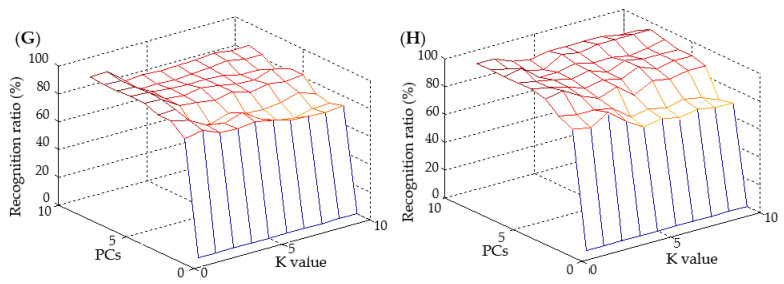
Application of composite films for pork freshness: (**A**) Photos of fresh pork packaged by composite films incorporated with red cabbage anthocyanin; (**B**) Changes in pH value of pork treated with different packaging during storage; (**C**) Changes in TBARS value of pork treated with different packaging during storage; (**D**) Changes in the total bacterial count of pork treated with different packaging during storage; (**E**) Changes in the TVB-N value of treated pork and the color of PSR 12% film with varied packaging throughout storage; (**F**) Qualitative discrimination of pork freshness: Principal component scatter plot; Discrimination rates of Training set by KNN (**G**) PSR 6%, (**H**) PSR 12%.

The control group utilized 10 g of fresh pork, possessing a consistent appearance, and was cut into approximately 10 g portions. In Figure 4A, the pork should be wrapped in CS/CSR6%/CSR12%. According to requirement, the pork must be enclosed in a ziplock bag to protect it from the surroundings before being stored in a refrigerator at a temperature of 4 °C. During the first ten days, a total of twenty pork samples were taken every two days. These samples were then analyzed for pH levels, lipid peroxide values, total viable count (TVC), and total volatile basic nitrogen (TVB-N) concentrations. In all, 120 samples were evaluated.

#### 3.3.3. Antioxidant and Antibacterial Properties

As demonstrated in Figure 4B, the pH of pork in the different treatment groups initially decreased and subsequently increased during the initial storage period [40]. After four days of storage, the pH of pork wrapped in various treatment groups was lower than that of the control group. This can be attributed to the CS film acting as a barrier to CO_2_, O_2_, and bacteria, thus limiting their capacity to degrade proteins into alkaline compounds. The pH decreased with the CSR film, which may have caused red cabbage anthocyanin to infiltrate the sample. On the 8th day, the pork packaged in the CS film became sub-fresh, while the pork packaged in the CSR film remained fresh.

Malondialdehyde is generated as a result of the oxidation of unsaturated fatty acids in meats [41]. A TBARS value less than 0.5 mg MDA/kg indicated the freshness of the pork [4]. As shown in Figure 4C, as time increased, the TBARS values for both the control group and the treated group of packaged pork increased. The TBARS value of pork packaged in the CS film was lower than that of the control group, but no significant difference was observed (*p* > 0.05). This can be attributed to the isolation of O_2_ by the CS film due to its excellent hydrophobic and gas barrier properties. The TBARS test result for pork packaged in the CSR film was higher due to the highest absorption peak of red cabbage anthocyanin at 520 nm, which had a more pronounced influence on the test outcomes as it penetrated the pork. The increase in TBARS values for pork packaged in CSR film was relatively lower compared to CS, attributed to the antioxidant activity of red cabbage anthocyanins in the film, which effectively delayed the lipid oxidation of the pork.

Total viable count (TVC) is an indicator of the extent of meat spoilage and acidification [42]. The TVC limit is set at 6 Lg CFU/g according to the GB/T 9959.2-2008 [42] standard for the evaluation of pork quality. Figure 4D illustrates the initial colony count of fresh pork, which started at 3.99 Lg CFU/g and steadily increased throughout the storage period in all three groups.

The CS film, functioning as an effective oxygen barrier, demonstrated its ability to inhibit the growth and reproduction of aerobic microorganisms during the early stages of storage. The Total Viable Count (TVC) value of pork packaged with the CS film was marginally lower than that of the control group. On the 4th day, the TVC of the control group had risen to 6.43 Lg CFU/g, indicating a loss of freshness (TVC > 6.00 Lg CFU/g). However, the TVC values of pork packed with CSR (6%) and CSR (12%) films were 5.66 and 5.55 Lg CFU/g, respectively, suggesting that the pork remained in a sub-fresh grade. The results showed that when the red cabbage anthocyanin concentration increased, the overall number of colonies decreased, which was associated with the natural bacteriostatic activity of red cabbage anthocyanin in the film.

Proteins found in meat are highly susceptible to bacterial contamination. During spoilage, various volatile nitrogenous compounds, such as ammonia and amines, are generated [43]. Total Volatile Basic Nitrogen (TVB-N) levels serve as an indicator of freshness, with values less than 15 mg/100 g considered fresh, between 15 and 20 mg/100 g considered acceptable, and exceeding 20 mg/100 g indicating spoilage [44].

As shown in Figure 4E, it has been discovered that when the period of storage increases, the TVB-N of pork wrapped in the control group and the treatment group showed a clear upward trend. However, the presence of red cabbage anthocyanin in the samples results in its diffusion and partial dissolution of H^+^, leading to a decline in pH. Notably, the TVB-N levels in the CSR film group exhibit an initial decline. In comparison to the control group, the CSR film group exhibited significantly lower TVB-N levels (*p* ≤ 0.05). This phenomenon can be attributed to the bacteriostatic properties of red cabbage anthocyanin. In the control group, the TVB-N levels increased from 8.12 mg/100 g to 14.466 mg/100 g within the initial two days of storage and then exhibited a substantial accumulation, reaching 23.987 mg/100 g after 6 days. In contrast, the TVB-N levels in the PSR/6% and PSR/12% film groups were 18.66 and 15.40 mg/100 g, respectively. This disparity suggests that the latter values are within an acceptable range for pork samples. Therefore, it can be inferred that red cabbage anthocyanin exerts a discernible preservative effect on pork samples [45].

The concentration of volatile salt nitrogen from the degradation of proteins in pork causes the film’s color to shift [2]. As shown in Figure 4E, the color of the CSR 12% film changed from pink to green, reacting to the shift from fresh to spoiled pork.

#### 3.3.4. Monitoring Freshness of Pork

The acquisition of composite film color signals including a total of 9 variables: *L*, *a*, *b*, *H*, *S*, and *V*, as well as *R*, *G*, and *B* values, is essential for the development of prediction models. According to the results of principal component analysis (PCA), films containing red cabbage anthocyanidin concentrations of 6% and 12% effectively distinguish between different stages of pork storage. In Figure 4F, the PCA diagram illustrates an example with a 12% concentration of red cabbage anthocyanidin, where the first, second, and third principal components contributed 78.83%, 17.72%, and 3.44%, respectively. The pork samples were classified into three categories based on their freshness: fresh (0–2 days), sub-fresh (3–5 days), and spoiled (6–7 days). To achieve precise differentiation rates, further analysis employed the K-nearest neighbor (KNN) algorithm [46]. Forty-five pork samples were randomly selected from the freshness and sub-freshness grades, and an additional 30 samples were drawn from the spoilage grade, resulting in a total of 120 samples. The remaining 40 samples were divided into both a training and a prediction set. Table S2 shows that the Lab model exhibited the highest recognition accuracy among the three color space models, and with K = 1, the number of variables is 3. The recognition rates of the KNN model on the correction set at CSR 6% and CSR 12% are 93.3% (Figure 4G) and 97.51% (Figure 4H), respectively. This showed that films with a 12% red cabbage anthocyanidin concentration are better indicators of pork freshness.

## 4. Conclusions

Subsequently, we developed an intelligent active film designed to monitor and preserve the freshness of pork by utilizing anthocyanins. This study examined the performance considerations when using gelatin, sodium alginate, and carboxymethyl cellulose as film-forming substrates. Impacts, such as performance stability, were compared. The CSR film exhibited the highest antioxidant capacity, boasting a DPPH free radical scavenging rate of 95.79%. The GCR film demonstrated the most potent inhibitory effect against *S. aureus*, *E. coli,* and *B. subtilis*. The CSR film exhibited the highest sensitivity to NH_3_ and maintained the best stability. Upon the addition of red cabbage anthocyanidin, the cross-linking in GS and GC films was disrupted, while the structure of the CS film became more compact, uniform, and better integrated. The CS film proved to be the most suitable substrate material. Subsequently, the concentration of red cabbage anthocyanidin added was also examined. At a concentration of 12% (*w*/*w*) of red cabbage anthocyanidin, the active film demonstrated the most effective freshness preservation and indication properties. The recognition rate of the KNN model on the correction set reached 97%. The films described in this study possess significant practical utility within the food industry, playing a pivotal role in guaranteeing the safety of pork and extending its shelf life.

## Figures and Tables

**Table 1:** Color and opacity of composite films with different mixed substrates.

Samples	*L*	*a*	*b*	Δ*E*	Opacity	Appearance
GS	85.35 ± 0.42 ^a^	−1.63 ± 0.12 ^b^	10.00 ± 0.65 ^b^	7.04 ± 0.66 ^d^	0.84 ± 0.05 ^d^	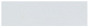
GSR	46.36 ± 2.66 ^c^	−8.14 ± 0.55 ^d^	4.48 ± 1.46 ^d^	43.11 ± 2.10 ^b^	3.24 ± 0.26 ^a^	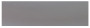
GC	85.74 ± 0.70 ^a^	−1.73 ± 0.07 ^b^	7.05 ± 0.71 ^c^	4.56 ± 0.82 ^e^	0.51 ± 0.21 ^e^	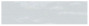
GCR	54.94 ± 0.52 ^b^	−3.81 ± 0.43 ^c^	19.04 ± 1.69 ^a^	34.38 ± 0.62 ^c^	2.43 ±0.09 ^b^	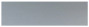
CS	86.83 ± 0.63 ^a^	−0.94 ± 0.26 ^b^	2.06 ± 0.06 ^e^	1.87 ± 0.42 ^f^	2.20± 0.06 ^c^	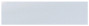
CSR	54.52 ± 1.40 ^b^	45.04 ± 1.52 ^a^	−4.91 ± 0.28 ^f^	58.23 ± 1.66 ^a^	2.60 ± 0.17 ^b^	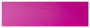

The mean ± standard deviation of the values in the table are given, along with the significance of the difference analysis using the same index of various films. Different lowercase letters (a, b, c, d, e, f) indicate significant differences (*p* < 0.05).

**Table 2:** Mechanical properties, WVP, MC and WS of composite films.

Samples	Thickness/μm	TS/MPa	EB/%	PS/N	MC/%	WS/%	WVP/10^−11^ (g m^−1^s^−1^ Pa^−1^)
GS	83.60 ± 5.47 ^b^	13.09 ± 5.60 ^c^	54.32 ± 2.09 ^bc^	4.01 ± 0.27 ^c^	29.36 ± 0.09 ^a^	27.39 ± 2.36 ^a^	3.08 ± 0.04 ^c^
GSR	96.80 ± 1.96 ^b^	18.70 ± 3.52 ^c^	48.17 ± 6.20 ^c^	3.08 ± 0.57 ^c^	28.81 ± 0.66 ^a^	28.04 ± 0.11 ^a^	5.70 ± 0.11 ^bc^
GC	98.03 ± 3.19 ^ab^	69.85 ± 1.51 ^b^	101.09 ± 2.58 ^a^	6.01 ± 0.28 ^b^	30.09 ± 1.17 ^a^	18.32 ± 1.01 ^b^	6.47 ± 0.24 ^bc^
GCR	104.20 ± 3.01 ^a^	94.72 ± 2.09 ^a^	83.50 ± 2.89 ^ab^	11.19 ± 0.34 ^a^	21.67± 1.52 ^a^	22.69 ± 0.94 ^b^	7.50 ± 0.02 ^b^
CS	82.06 ± 1.55 ^b^	47.76 ± 6.42 ^b^	43.70 ± 2.04 ^c^	1.50 ± 0.92 ^c^	32.69 ± 0.38 ^a^	20.60 ± 0.66 ^b^	6.02 ± 0.60 ^bc^
CSR	92.07 ± 3.68 ^ab^	47.79 ± 8.97 ^b^	88.71 ± 9.91 ^b^	1.92 ± 0.37 ^c^	16.52 ± 1.26 ^b^	27.38 ± 1.31 ^a^	11.69 ± 0.23 ^a^

The mean and standard deviation of the values in the table are given, along with the significance of the difference analysis using the same index of various films. Various lowercase letters (a, ab, b, bc, c) indicate significant differences (*p* < 0.05).

## Data Availability

Data is contained within the article or Supplementary Materials.

## Supplementary Materials

The following supporting information can be downloaded at: https://www.mdpi.com/article/10.3390/foods12244520/s1, Figure S1: The change color of film; Table S1: Inhibitory zone diameters of film forming solution against *E.coli*, *B.subtilis* and *S.aureus*; Table S2: Comparation of KNN model by different color features.

## Abbreviations

Gelatin, G; Sodium Alginate, SA; Carboxymethyl Cellulose, CMC; Red cabbage anthocyanin, R; total volatile base nitrogen, TVBN; total viable count, TVC; tensile strength, TS; elongation at break, EB; Moisture content, MC; water solubility, WS; Water vapor permeability, WVP; optical density, OD; thiobarbituric acid, TBA; Gelatin-Sodium Alginate, GS; Gelatin-Sodium Alginate-red cabbage anthocyanin, GSR; Gelatin-Carboxymethyl Cellulose, GC; Gelatin-Carboxymethyl Cellulose-red cabbage anthocyanin, GCR; Carboxymethyl Cellulose -Sodium Alginate, CS; Carboxymethyl Cellulose-Sodium Alginate-red cabbage anthocyanin, CSR.
