# Supplementary material for: Preparation of a Dual-Functional Active Film Based on Bilayer Hydrogel and Red Cabbage Anthocyanin for Maintaining and Monitoring Pork Freshness
Source: Foods. 2023 Dec 18;12(24):4520. doi: 10.3390/foods12244520 (PMC10742916; doi:10.3390/foods12244520)
Supplement: Supplementary file 1 [file foods-12-04520-s001.zip › foods-2697937-supplementary.pdf]

Supplementary material

Preparation of Dual-Functional Active Film based on Bilayer Hydrogel and Red Cabbage Anthocyanin for Maintaining and Monitoring Pork Freshness

Huang Xiaowei[a b c] Zhao Wanying[a] Li Zhihua[a*] Zhang Ning[a] Wang Sheng[a] Shi Jiyong[a b] Zhai Xiaodong[a] Zhang Junjun[a] Shen Tingting[a]

[1] School of Food and Biological Engineering, School of Agricultural Equipment Engineering, Jiangsu University, 301 Xuefu Rd., Zhenjiang 212013, China; huangxiaowei@ujs.edu.cn (X.H.); 15506509723@163.com (W.Z.); zhangning980409@163.com (N.Z.); 19825822627@163.com (S.W.); shi-jiyong@ujs.edu.cn (J.S.);

zhai_xiaodong@ujs.edu.cn (X.Z.); jjzhang95@126.com (J.Z.); shentingtingstt@ujs.edu.cn (T.S.)

[2] College of Food Science and Engineering, Nanjing University of Finance and Economics/Collaborative Innovation Center for Modern Grain Circulation and Safety, 128 North Railway Street, Gulou District, Nanjing 210023, China

3. Focusight (Jiangsu) Technology Co., Ltd., No. 258-6 Jinhua Road, Wujin Economic Development Zone, Changzhou 213146, China

* Correspondence: lizh@ujs.edu.cn

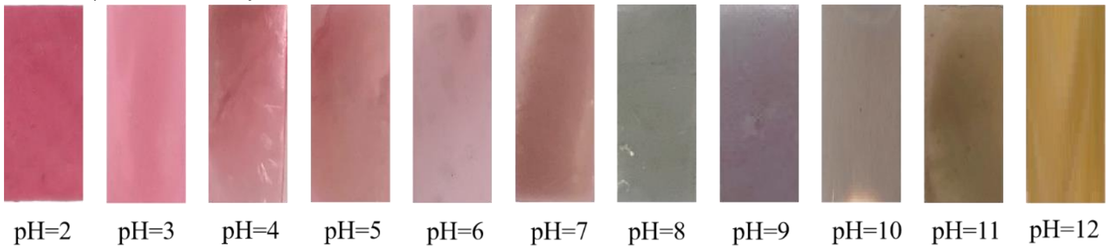

Figure S1 The change color of film

Table S1. Inhibitory zone diameters of film forming solution against *E.coli*、*B.subtilis* and *S.aureus*

Sample	*E.coli*/mm	*B.subtilis*/mm	*S.aureus*/mm
GS	7.53±0.22[b]	7.62±0.07[b]	7.20±0.09[b]
GSR	8.55±0.09[a]	7.82±0.11[b]	7.42±0.12[b]
GC	8.64±0.26[a]	7.47±0.31[b]	8.47±0.03[a]
GCR	8.75±0.30[a]	8.07±0.62[a]	8.30±0.21[a]
CS	6.33±0.81[c]	6.90±0.02[c]	6.41±0.52[c]
CSR	6.67±0.23[c]	6.27±0.13[c]	6.70±0.10[c]

Different letters in the same column denote significant differences between films (Duncan's test, $p < 0.05$).

Table S2. Comparation of KNN model by different color features

Characteristic variables	CSR 6%			CSR 12%		
	PC number	*K* value	recognition rate of the training set	PC number	*K* value	recognition rate of the training set
RGB	3	3	85.0%	3	3	79.1%
Lab	3	1	97.5%	3	1	92.5%
HSV	3	3	92.5%	3	3	84.1%
**Fusion features**	**6**	**1**	**97.5%**	**6**	**1**	**93.3%**
